# Supplementary figures and images for: A novel air quality monitoring and improvement system based on wireless sensor and actuator networks using LoRa communication
Source: PeerJ Comput Sci. 2021 Sep 16;7:e711. doi: 10.7717/peerj-cs.711 (PMC8459792; doi:10.7717/peerj-cs.711)

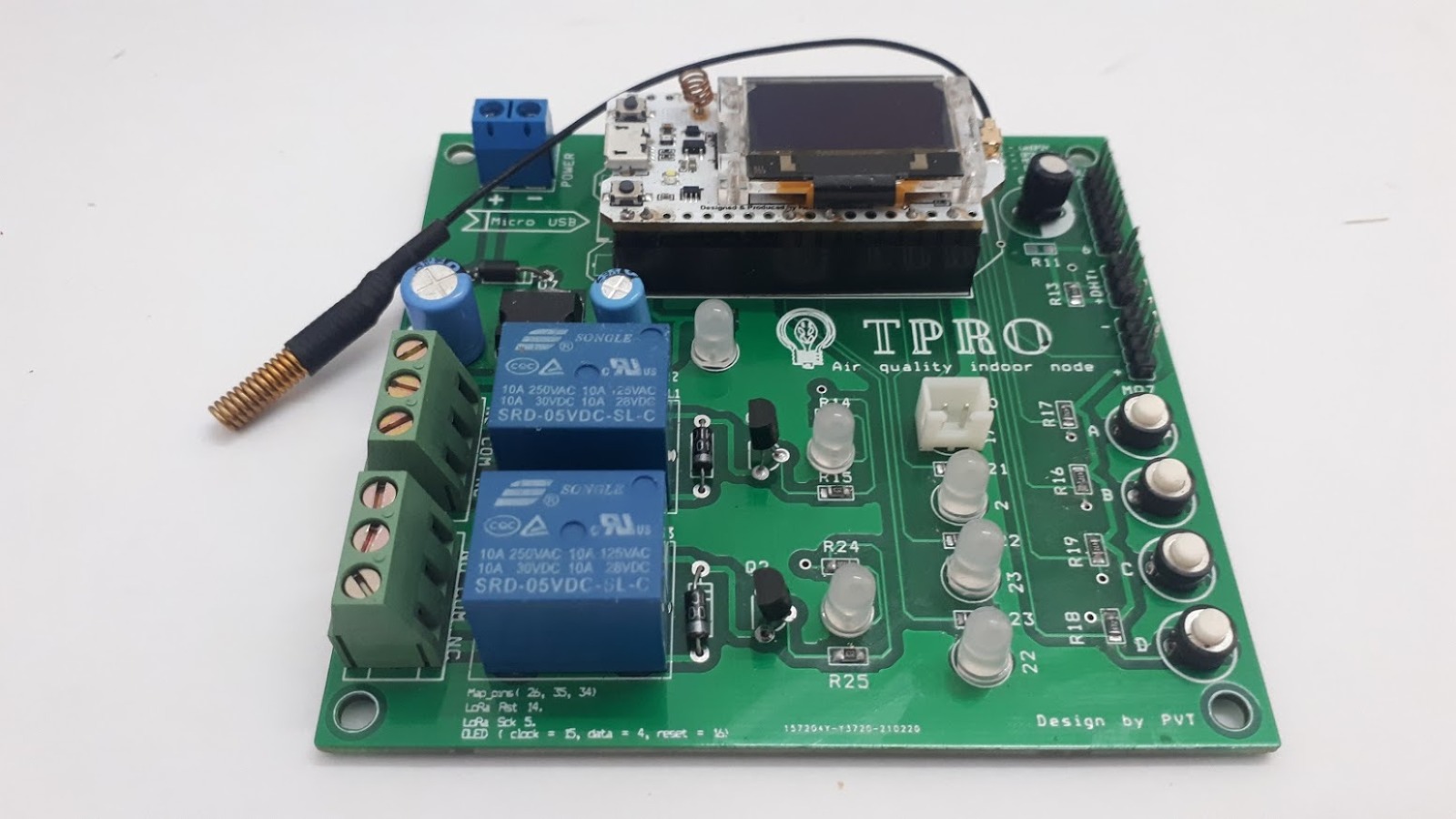

Supplement: Supplemental Information 4 [file peerj-cs-07-711-s004.jpg]

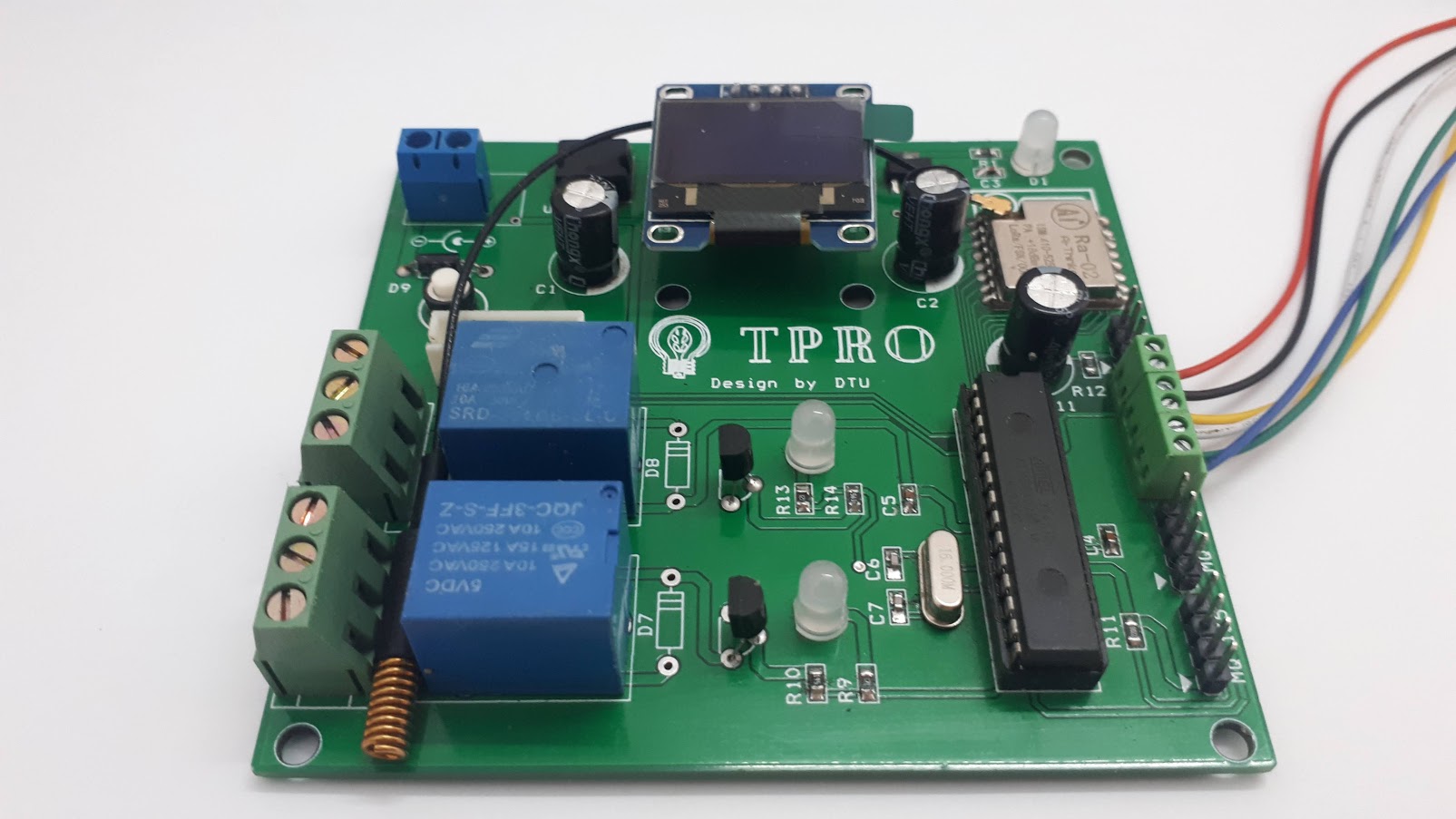

Supplement: Supplemental Information 6 [file peerj-cs-07-711-s006.jpg]

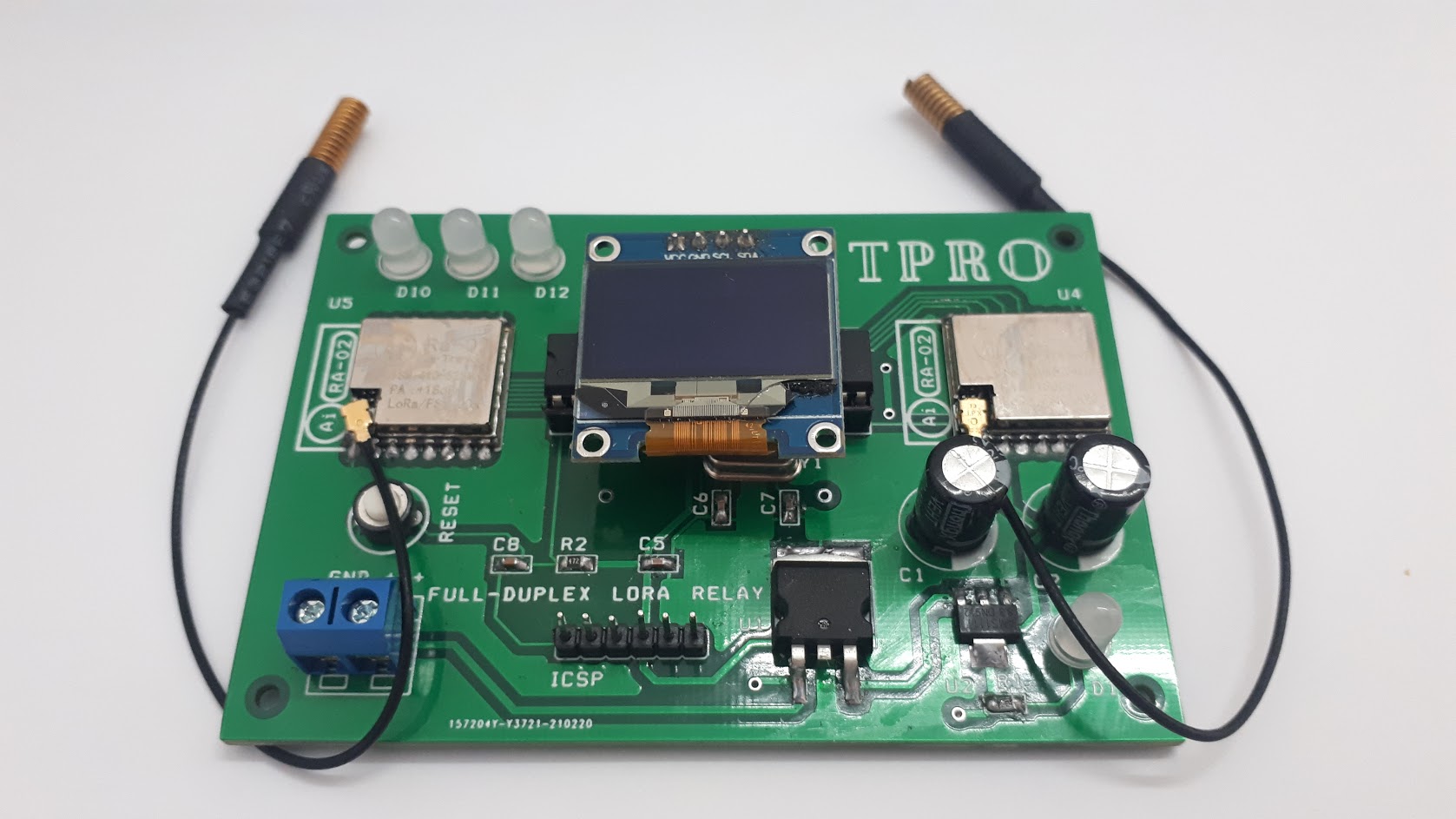

Supplement: Supplemental Information 7 [file peerj-cs-07-711-s007.jpg]
